# Supplementary material for: Prioritizing plant defence over growth through WRKY regulation facilitates infestation by non-target herbivores
Source: eLife. 2015 Jun 17;4:e04805. doi: 10.7554/eLife.04805 (PMC4491539; doi:10.7554/eLife.04805)
Supplement: Supplementary file 1. — (A) Primers used for cloning of full-length or partial cDNAs of target genes in this study. (B) Primers and probes used for QRT-PCR of target genes. DOI: http://dx.doi.org/10.7554/eLife.04805.019 [file elife04805s001.doc]

**Supplementary file 1**

**Supplementary file 1A. Primers used for cloning of full-length or partial cDNAs of target genes in this** study

| **Gene** | **Sequence (5’-3’)** | **Purpose** |
| --- | --- | --- |
| OeWRKY70-F | TTCTCCTCCGTCTCCGATGA | oeWRKY70 construct |
| OeWRKY70-R | ATCATCAAAACAGCAGCGGC | oeWRKY70 construct |
| irWRKY70-F1 | GTCGACCTTCTCCAGCCTGCTCAGTG | irWRKY70 construct |
| irWRKY70-R1 | GGTACCTGGTCGGTTCTTGGTAGCTC | irWRKY70 construct |
| irWRKY70-F2 | ATGAGCTCCTTCTCCAGCCTGC | irWRKY70 construct |
| irWRKY70-R2 | TAACTAGTTGGTCGGTTCTTGG | irWRKY70 construct |
| WRKY70-peGFP-F | AGTCGACATGACCGCCGCGCCG | localization |
| WRKY70-peGFP-R | AAGGATCCTCAAACAGCAGCGGCTGC | localization |
| N-WRKY70-F | AATATGTCGACATGACCGCCGCGCCGGGG | BiFC |
| N-WRKY70-R | ACTATGGATCCAAACAGCAGCGGCTGCTCAA | BiFC |
| MPK3-C-F | AGGCCGAATTCATGGACGGGGCGCCGGTGG | BiFC |
| MPK3-C-R | ACGTTGGATCCCGTACCGGATGTTTGGGTTCA | BiFC |
| MPK6-C-F | ATGTTGTCGACGGATGGACGCCGGGGCGCA | BiFC |
| MPK6-C-R | ACGTTGGATCCCCTGGTAATCAGGGTTGAA | BiFC |
| PET28b-MKK4-F | GTACTCATATGATGCGACCGGGCGGGCCGC | Protein expression |
| PET28b-MKK4-R | GTACAGCGGCCGCTGACGGAGGCGGTGCGA | Protein expression |
| 6myc-DC-MKK4-F | CACCATGCGACCGGGCGGGCCGC | Transient expression |
| 6myc-DC-MKK4-R | TGACGGAGGCGGTGCGAGGG | Transient expression |
| PET32-WRKY70-F | AAGGATCCATGACCGCCGCGCCGGG | Protein expression |
| PET32-WRKY70-R | TGTCGACGAAACAGCAGCGGCTGCT | Protein expression |
| pGEX-MPK3-F | AAGGATCCATGGACGGGGCGCCGGT | Protein expression |
| pGEX-MPK3-R | AGTCGACCTAGTACCGGATGTTTGGG | Protein expression |
| MPK3-YFP-F | CGCCTCCTAGGAATGGACGGGGCGCCGGTG | Transient expression |
| MPK3-YFP-R | AGTAGCCCGGGAGTACCGGATGTTTGGGTTC | Transient expression |
| pGEX-MPK6-F | AAGGATCCATGGACGCCGGGGCGCA | Protein expression |
| pGEX-MPK6-R | AGTCGACCTACTGGTAATCAGGGTTG | Protein expression |
| MPK6-YFP-F | TAACTCCTAGGAATGGACGCCGGGGCGCAGC | Transient expression |
| MPK6-YFP-R | AGTAGCCCGGGACTGGTAATCAGGGTTGAACG | Transient expression |
| 1391-Promoter_WRKY70_-F | GCGGCCTGCAGCCTCATTAGAGTGAACGCACAT | GUS reporter |
| 1391-Promoter_WRKY70_--R | TCGACGTCGACGCAGAGTAGGATGCCAGGAT | GUS reporter |
| GBKT7-WRKY70-F | AAGGATCCGTATGACCGCCGCGCCG | Y1H |
| GBKT7-WRKY70-R | AGTCGACGAAACAGCAGCGGCTGC | Y1H |

**Supplementary file 1B.** Primers and probes used for QRT-PCR of target genes

| Gene | Tigr ID | Primer (5’-3’) | Probe (5’-3’) |
| --- | --- | --- | --- |
| *OsMPK3* | Os03g17700 | FP: CGACTTCGAGCAGAAGGCTCTA  RP: GTTCATCTCGATCGCTTCGTT | ACGAGGACCAAATGAAGCAGCTGAT |
| *OsWRKY53* | Os05g27730 | FP: AACGGCTGCTCCATGAAGAA  RP: TTGTGTGCGCCCTTGTAGAC | CTCGCCGACGGCCGCATC |
| *OsWRKY70* | Os05g39720 | FP: CCGCTGCTGTTTTGATCATCT  RP: GGAGCTAAGCTAACTCACTCCACA | ATCGGGCCGTCAATTTGATCAGCA |
| *OsHI-LOX* | Os08g39840 | FP: CCGAGCTTGACGCGAAGA  RP: GATCGTCGTCGTCCACATTGT | CGGGAAGGAGAAAGCAACTGTGCG |
| *OsACS2* | Os04g48850 | FP: CACCCCGAGGCATCCAT  RP: ATTGGCGATCCTCTTGAACTG | TGCACACCGGAGGGCGTCT |
| *OsACT* | Os03g50885 | FP: TGGACAGGTTATCACCATTGGT  RP: CCGCAGCTTCCATTCCTATG | CGTTTCCGCTGCCCTGAGGTCC |
| *OsMPK6* | Os06g06090 | FP: CGCACGCTCAGGGAGATC  RP: GGTATGATATCCCTTATGGCAACAA | CTCCGCCACATGGACCACGAGAA |
| *OsWRKY24* | Os01g61080 | FP: AAGAGATGGAGGAAAGACGGTG  RP: TGTCGATGTCGCTCATGGTT | AGGGGATCTCCATGGCTGGCAA |
| *OsAOS2* | Os03g12500 | FP: TGCCCATGATCATCGAGGAT  RP: TGTAGTCGGAGCTGATGAGGAA | CTCCTCCACACGCTGCCGCTG |
| *OsICS1* | Os09g19734 | FP: TCTGCGATGATGTGGTTGTC  RP: GCTCGGATGAAGAGTATTTAGGA | CATCCCAGCAAGGCCCTTCG |
| *OsGA20OX7* | Os08g44590 | FP: GACTACGGCTTCCTCACGCT  RP: TTCACCTTCACCCTGTGCAG | CGTTGACGACGAAGGAGCCCG |
